# Effects of Organic Amendments on Microbiota Associated with the *Culex nigripalpus* Mosquito Vector of the Saint Louis Encephalitis and West Nile Viruses

**DOI:** 10.1128/mSphere.00387-16

**Published:** 2017-02-01

**Authors:** Dagne Duguma, Michael W. Hall, Chelsea T. Smartt, Josh D. Neufeld

**Affiliations:** a Florida Medical Entomology Laboratory, IFAS, University of Florida, Vero Beach, Florida, USA; b Faculty of Computer Science, Dalhousie University, Halifax, Nova Scotia, Canada; c Department of Biology, University of Waterloo, Waterloo, Ontario, Canada; University of Wisconsin, Madison

**Keywords:** aquatic chemistry, bacteria, disease vectors, food web, life stages, microbiome, mosquito, pollution

## Abstract

Pollution from nutrients in aquatic habitats has been linked to increases in disease vectors, including mosquitoes and other pestiferous insects. One possibility is that changes in mosquito microbiomes are impacted by nutrient enrichments and that these changes affect various traits, including larval development, susceptibility to larval control agents, and susceptibility of the adult mosquitoes to pathogens. We tested this hypothesis using field mesocosms supplemented with low- and high-organic-nutrient regimens and then sampled microbial communities associated with the naturally colonizing *Culex nigripalpus* mosquito vector. By high-throughput sequencing of 16S rRNA gene sequences, we found no significant differences in overall microbial communities associated with sampled mosquitoes, despite detecting discernible differences in environmental variables, including pH, dissolved oxygen, and nutrient amendments. Nevertheless, indicator species analysis revealed that members of the *Clostridiales* were significantly associated with mosquitoes that originated from high-nutrient enrichments. In contrast, members of the *Burkholderiales* were associated with mosquitoes from the low-nutrient enrichment. High bacterial variability associated with the life stages of the *C. nigripalpus* was largely unaffected by levels of nutrient enrichments that impacted larval microbial resources, including bacteria, ciliates, and flagellates in the larval environments.

**IMPORTANCE** Mosquito microbiota provide important physiological and ecological attributes to mosquitoes, including an impact on their susceptibility to pathogens, fitness, and sensitivity to mosquito control agents. *Culex nigripalpus* mosquito populations transmit various pathogens, including the Saint Louis and West Nile viruses, and proliferate in nutrient-rich environments, such as in wastewater treatment wetlands. Our study examined whether increases in nutrients within larval mosquito developmental habitats impact microbial communities associated with *C. nigripalpus* mosquitoes. We characterized the effects of organic enrichments on microbiomes associated with *C. nigripalpus* mosquitoes and identified potential bacterial microbiota that will be further investigated for whether they alter mosquito life history traits and for their potential role in the development of microbial-based control strategies.

## Abstract (precis)

Mosquito microbiota provide important physiological and ecological attributes to mosquitoes, including an impact on their susceptibility to pathogens, fitness, and sensitivity to mosquito control agents. *Culex nigripalpus* mosquito populations transmit various pathogens, including the Saint Louis and West Nile viruses, and proliferate in nutrient-rich environments, such as in wastewater treatment wetlands. Our study examined whether increases in nutrients within larval mosquito developmental habitats impact microbial communities associated with *C. nigripalpus* mosquitoes. We characterized the effects of organic enrichments on microbiomes associated with *C. nigripalpus* mosquitoes and identified potential bacterial microbiota that will be further investigated for whether they alter mosquito life history traits and for their potential role in the development of microbial-based control strategies.

## INTRODUCTION

Nutrient pollution due to excess use of nitrogen and phosphorus can lead to an increased risk of vector-borne diseases (1–5). Previous field studies reported an increase in the abundance of mosquito vectors with an increase in nutrients in mosquito larval developmental sites (6–11). Moreover, increases in nutrient enrichments in mosquito larval developmental sites have been known to reduce the efficacy and persistence of larval control agents (12–15). Higher doses of mosquito larvicides are often required to have a significant reduction in mosquito larval population in organic-rich environments, suggesting a higher economic cost of mosquito control in polluted environments than in less polluted environments.

Nutrient enrichments are generally thought to cause changes in microbial communities, including bacteria, ciliates, flagellates, microalgae, and rotifers that are considered essential for larval mosquito development (9, 10, 16), and could alter aquatic food webs in an unpredictable manner. For example, elevation of nutrients in freshwater streams altered invertebrate predator-prey relationships from linear to curvilinear (17, 18). However, the underlying mechanisms of nutrients and mosquito vector interactions are not fully understood.

*Culex nigripalpus* Theobald is a major vector of Saint Louis encephalitis virus and is responsible for transmitting other pathogens in the southeastern United States, including West Nile virus (19, 20). *Culex nigripalpus* is among the dominant mosquito species found during early succession stages of newly developed aquatic habitats, including following rainfall and in polluted treatment wetlands (21–24). Significant genetic variations are known to exist among various populations of *C. nigripalpus* (25). Variations in abundance among larval developmental sites (21), susceptibility to infection and transmission of pathogens among geographic populations (26), and susceptibility to organophosphate-based pesticides (27) have also been documented. However, little is known about whether nutrient-mediated changes, including water quality variables and microbial consortia found in larval developmental habitats, can influence the mosquito-associated microbiomes for *C. nigripalpus* developing in different environments.

Bacteria associated with mosquitoes are found to be crucial sources of nutrition for successful larval development (28–31), affect mosquito susceptibility by various pathogens (32–35), impact resistance to pesticides (36, 37), and influence mosquito oviposition (38). As a result, understanding the effects of nutrients on microbial communities associated with mosquitoes is critical for disentangling the underlying causes of variability in disease transmission, variations in mosquito production among various aquatic habitats, and lack of susceptibility to pesticides. In addition, this knowledge is important for the development of novel microbial (e.g., *Wolbachia*) mosquito control strategies. We hypothesized that different nutrient regimens in larval habitats impact microbial communities associated with mosquitoes developing during the succession of these habitats. In order to test this hypothesis, we characterized microbiota associated with *C. nigripalpus* developing in two different resource (nutrient) regimens under natural field conditions. In addition, we characterized microbial communities in different life stages of *C. nigripalpus* to identify potential symbionts associated with all life stages.

## RESULTS

### Environmental variables in the water column.

**FIG 1:**
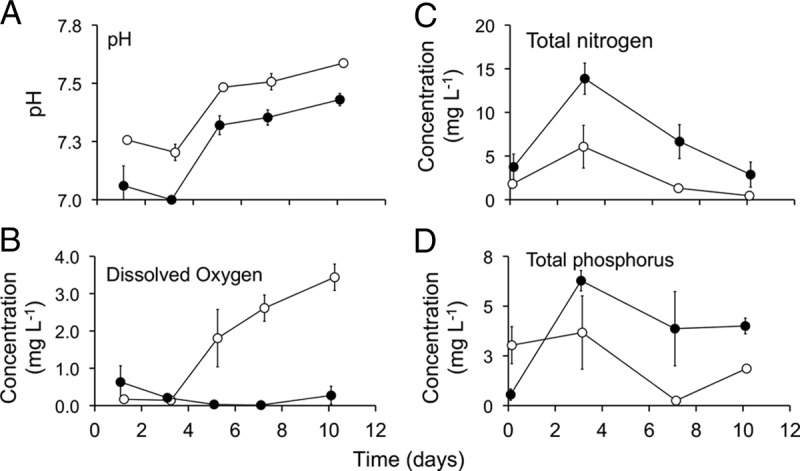
Water quality parameters. Mean ± SE (*n* = 3) pH, dissolved oxygen, total nitrogen, and total phosphorus in water of the low (○)- and high (●)-nutrient treatments of differentially treated larval habitats (mesocosms). The *x* axis represents time in days after mesocosms were uncovered and *Culex* mosquitoes laid egg rafts on water. The *y* axis denotes the concentration or values of water quality parameters. For example, the mesocosms were exposed to egg-laying female mosquitoes on 2 November 2015.

The water quality indicators differed significantly between two contrasting larval environments (i.e., high and low nutrients) in outdoor experimental mesocosms (Fig. 1). Significantly lower pH values (Fig. 1A [*F*_1, 4_ = 55, *P* = 0.002]) and dissolved oxygen concentrations (Fig. 1B [*F*_1, 4_ = 25, *P* = 0.007]) were found in high-nutrient mesocosms. In contrast, higher chemical oxygen demand (COD) was found in the high-nutrient mesocosms (mean ± standard error [SE], 239 ± 45.8 mg/liter) compared to the low-nutrient mesocosms (143 ± 8 mg/liter) on day 7, although the difference was not statistically significant (*P* = 0.107). A similar trend but lower concentration was observed on day 9, with 195 ± 19.8 mg/liter and 150 ± 18.6 mg/liter in the high- and low-nutrient treatments, respectively.

A higher concentration of total nitrogen (significance tested after Bonferroni correction) was also found in high-nutrient treatments than in the low-nutrient treatments and was variable across time (Fig. 1C [*F*_1, 4_ = 5.8, *P* = 0.07]). Similarly, a higher total phosphorus concentration was found in the high- than in the low-nutrient-treated mesocosms (Fig. 1D [*F*_1, 4_ = 4.7, *P* = 0.12]). Temperature and light intensity in the water column varied temporally but were relatively uniform among mesocosms and were not significantly affected by nutrient enrichments (data not shown).

**FIG 2:**
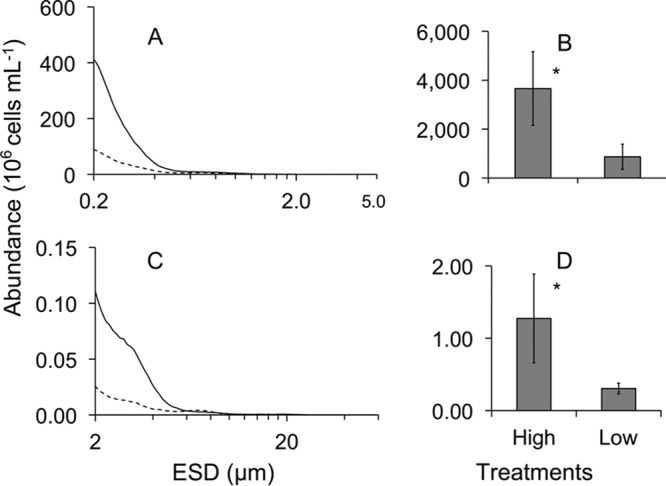
Heterotrophic and autotrophic particle (cell) abundance in water column. Small (0.2- to 1.999-μm equivalent spherical diameter [ESD]) (A) and large (2- to 60-μm ESD) (C) particle size distribution in high (solid lines)- and low (dashed lines)-organic-nutrient-enriched mesocosms, and mean ± SE total particle abundance (*n* = 3) of small (B) and large (D) particle sizes in water of low- and high-nutrient treatments on day 0.

The abundances of small (0.2- to 1.999-μm equivalent spherical diameter [ESD]) and large (2- to 60-μm ESD) organic particles differed significantly between the two treatments on the first day that the mesocosms were exposed to egg-laying female mosquitoes (Fig. 2). Mean total abundance of small particles was significantly greater in high-nutrient treatments than low-nutrient treatments (Fig. 2A and B [*F*_1, 15_ = 10.5, *P* = 0.005]). Similarly, the mean abundance of large particles was approximately 5-fold greater in the high-nutrient treatments than in the low-nutrient treatments (Fig. 2C and D [*F*_1, 15_ = 14.6, *P* = 0.002]).

### Microeukaryote abundance.

**FIG 3:**
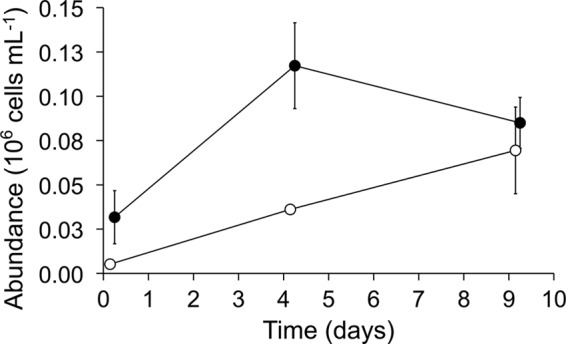
Microeukaryote abundance in water column. Mean ± SE (*n* = 3) abundance of ciliate protists, flagellates, and rotifers in the mesocosms with high (●)- and low (○)-nutrient treatments on days 0, 4, and 9 after egg laying by mosquitoes. Error bars not seen are contained within the symbols.

Microscopic examination of water samples for microeukaryotes (i.e., ciliates, flagellates, and rotifers) revealed a significant difference in the combined abundance of these microorganisms between the two treatments (Fig. 3 [*F*_1, 16_ = 24.1, *P* = 0.0002]) and varied across time (*F*_2, 15_ = 4.3, *P* = 0.04). Significantly greater numbers of microeukaryotes were found in high-nutrient treatments on days 0 and 7, but that difference decreased by day 9.

### Mosquito larva abundance.

Microscopic examination of the dipper samples revealed no significant differences in total *Culex* larval abundance between the two treatments at either 5 days or 7 days after the mesocosms were exposed to naturally occurring mosquitoes (*F*_1, 4_ = 0.006, *P* = 0.9). The mean ± SE number of *C. nigripalpus* larvae found in the low-nutrient treatments was 41 ± 10, compared with the high-nutrient treatments, with 32 ± 27 larvae per dip sample. Very few individuals of the southern house mosquito *Culex* *quinquefasciatus* Say were observed on this sampling date in the low (average; <1 larvae)- and high (~4 larvae per dipper sample)-nutrient treatments. A similar trend was observed a week after uncovering the mesocosms (data not shown).

### Diversity of bacterial communities in mosquitoes from different nutrient treatments.

**FIG 4:**
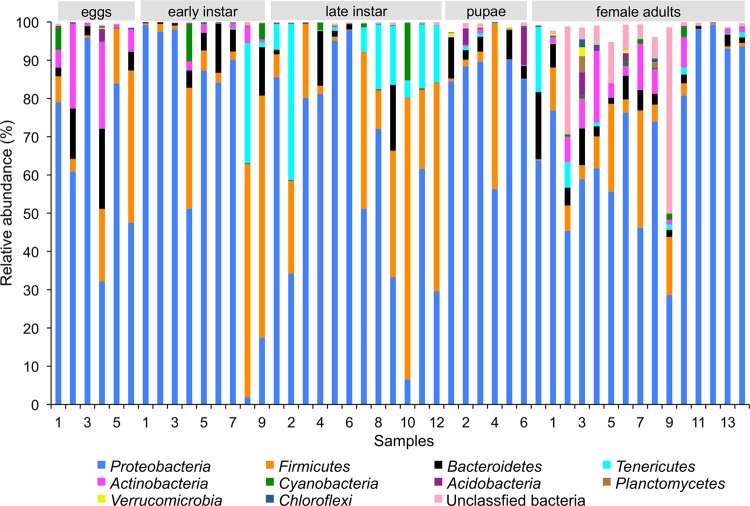
Dominant bacterial phyla found in different stages. Shown are bacterial phyla in eggs, early and late larval instars, pupae, and newly emerged female adults of *Culex nigripalpus* developed under the field conditions. Only phyla with an average abundance of >0.1% were included. Other unclassified sequences accounted for 0.3%. *Archaea* and an additional 16 phyla accounted for <0.1%. Egg samples 1, 4, and 5, early instar samples 7, 10, 11, and 18, late instar samples 14, 18, 20, 41, 42, 46, and 47, pupa samples 20 and 38, and female adult samples 32 to 36 were taken from high-nutrient regimens. The remaining 27 samples were derived from low-nutrient regimens.

A total of 4,859,297 sequences in 1,751 operational taxonomic units (OTUs) were generated from 48 mosquito samples, including 6 half-egg rafts, 9 early and 12 late instar larvae, 7 pupae, and 14 newly (<1 day after eclosion) emerged non-blood-fed female adults of* C. nigripalpus*. Assembled and quality-checked sequences had a mean length of 416 bases with a mean overlap of 49.8 bases. On average, 83,408 sequences from egg rafts, 106,809 from early instar larvae, and 116,281 from late instar larvae, 89,430 from pupae, and 83,107 from female adults per sample were obtained. These were classified into 28 bacterial phyla with *Proteobacteria*, *Firmicutes*, *Bacteroidetes*, *Tenericutes*, *Actinobacteria*, and *Acidobacteria* dominating the bacterial phyla found associated with this mosquito species (Fig. 4). *Proteobacteria* accounted for nearly 70% of sequences, followed by *Firmicutes*, with 15% of all sequences. Approximately 56% of the sequences were identified as 240 genera, with *Arcobacter* (*Epsilonproteobacteria*: *Campylobacteraceae*) being the most abundant (29%) genus, followed by *Thorsellia* (7%). Few archaeal sequences (0.002%) were recovered from this mosquito species, and those were primarily from female adults.

**FIG 5:**
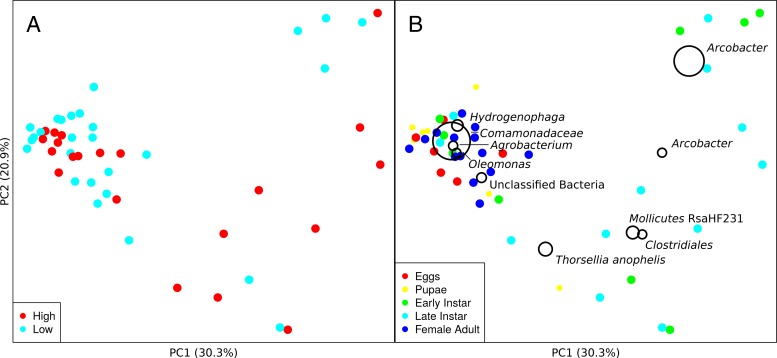
PCoA ordination based on the weighted UniFrac distance metric. Shown is ordination of microbial communities associated with *Culex nigripalpus* mosquitoes colored by either nutrient treatment level (A) or life stage (B). The effects of nutrients on microbial communities associated with the mosquitoes were not substantial (MRPP; *A* = 0.005, *P* = 0.08) between the two treatments. However, the microbiota in the different life stages differed weakly but significantly (MRPP; *A* = 0.08, *P* = 0.001). Large open circles indicate the 10 most abundant bacterial taxa associated with samples.

The principle coordinate (PCoA) ordinations based on weighted UniFrac measures revealed no significant differences in bacterial community composition found among mosquitoes developing in low- and high-nutrient treatments (Fig. 5A [multiresponse permutation procedure [MRPP; *A* = 0.005; *P* = 0.081]). The greatest variation (indicated by principal coordinate 1 [PC1]) detected among samples was attributed to the higher abundance of OTUs corresponding to *Arcobacter* (OTU 0) and an unidentified species of* Comamonadaceae* (OTU 2). *Arcobacter* (*Epsilonproteobacteria*) was mostly associated with larvae of *C. nigripalpus*, whereas the unidentified species in *Comamonadaceae* (*Betaproteobacteria*) was found associated with all life stages of *C. nigripalpus*, including egg rafts. Differences in bacterial communities among larval samples from low- and high-nutrient treatments were not significant (MRPP; *A* = 0.018; *P* = 0.084).

Indicator species analysis revealed that members of *Clostridiales* dominated bacterial communities associated with mosquitoes developing in high-nutrient regimens (indicator values = 0.5 to 0.8; *P* ≤ 0.01), whereas mosquitoes from low-nutrient treatments were enriched with *Burkholderiales* (see Table S1 in the supplemental material [indicator value = 0.48; *P* < 0.01]). Although differences among stages were apparent, indicator species analysis by life stages revealed that *Comamonadaceae* OTUs were strongly associated with all life stages, except pupal samples (see Table S1 and Fig. S1 in the supplemental material).

10.1128/mSphere.00387-16.1 TABLE S1  Indicator bacterial species (indicator value, ≥0.4; *P* < 0.05) associated with *Culex nigripalpus* Theobald developing in low- and high-organic-nutrient enrichments. Download TABLE S1, PDF file, 0.1 MB. Copyright © 2017 Duguma et al. 2017 Duguma et al. This content is distributed under the terms of the Creative Commons Attribution 4.0 International license.

10.1128/mSphere.00387-16.2 FIG S1  Relative abundance of the 16 most abundant (>1,000 sequences per sample) members of families recovered from different life stages of field-reared *Culex nigripalpus* mosquitoes. Others include 148 families (660,633 sequences) that had sequence abundance of <1,000 sequences per sample. The remaining 747 OTUs (692,057 sequences) were unidentified to families. Download FIG S1, EPS file, 0.3 MB. Copyright © 2017 Duguma et al. 2017 Duguma et al. This content is distributed under the terms of the Creative Commons Attribution 4.0 International license.

Nonmetric multidimensional scaling (NMDS) analysis based on Bray-Curtis distance measures revealed significant differences between microbiota samples that originated from different life stages of mosquitoes (Fig. 5B; see Fig. S2 in the supplemental material [MRPP; *A* = 0.08; *P* = 0.001]). Microbial samples from female adults were significantly separated from the immature stages and egg rafts of *C. nigripalpus*.

10.1128/mSphere.00387-16.3 FIG S2  Nonmetric multidimensional scaling (NMDS) plot of bacterial communities from different life stages of field-reared *Culex nigripalpus* mosquitoes. Download FIG S2, PDF file, 0.1 MB. Copyright © 2017 Duguma et al. 2017 Duguma et al. This content is distributed under the terms of the Creative Commons Attribution 4.0 International license.

Nine core taxa were found in all samples of all life stages of *C. nigripalpus*, including *Thorsellia anophelis* OTU 4, *Oleomanas* OTU 7, two unidentified species (OTUs 1 and 2) of *Comamonadaceae*, two *Hydrogenophaga* species (OTUs 18 and 1537), an unidentified species (OTU 71) of *Cyanobacteria*, an unidentified species (OTU 8) of *Firmicutes*, and an unidentified species (OTU 3) of *Tenericutes* (see Fig. S3 in the supplemental material).

10.1128/mSphere.00387-16.4 FIG S3  Relative abundance of the 22 most abundant (average abundance of ≥1,000 sequences per sample) OTUs recovered from different life stages of field-reared *Culex nigripalpus* mosquitoes. Others include OTUs with average abundance of <1,000 sequences per sample and include 1,729 OTUs with a total of 1,968,535 sequences. The lowest taxonomic classifications of OTUs are presented. Download FIG S3, EPS file, 0.3 MB. Copyright © 2017 Duguma et al. 2017 Duguma et al. This content is distributed under the terms of the Creative Commons Attribution 4.0 International license.

### Bacterial communities in egg rafts of *Culex nigripalpus*.

Bacterial sequences from egg rafts of *C. nigripalpus* grouped into 530 OTUs that were dominated by an unknown species (OTU 2) of *Comamondaceae* (35%), followed by *Agrobacterium* OTU 10 (12%) (see Table S2 and Fig. S3 in the supplemental material).

10.1128/mSphere.00387-16.5 TABLE S2  Indicator bacterial species (indicator value, ≥0.5; *P* < 0.05) associated with different life stages (egg to adults) of *Culex nigripalpus*. Download TABLE S2, PDF file, 0.1 MB. Copyright © 2017 Duguma et al. 2017 Duguma et al. This content is distributed under the terms of the Creative Commons Attribution 4.0 International license.

### Bacterial communities in immature stages of* Culex nigripalpus*.

An *Epsilonproteobacteria* member, *Arcobacter* (31%), and two OTUs corresponding to species in *Betaproteobacteria* (an unknown species in* Comamondaceae* [14%] and *Vogesella* [*Neisseriaceae*]) dominated bacterial communities associated with early larval instars. *Thorsellia anophelis* was also recovered from the early instar stage but in a much lower (<1%) proportion (see Table S3 in the supplemental material). Bacteria in late instar larvae were also dominated by *Arcobacter* (27%), *Thorsellia anophelis* (10.5%), and an unknown genus of *Mollicutes* (10%). *Hydrogenophaga* (14%), *Thorsellia* (11%), and an unknown species of *Comamondaceae* (10%) dominated bacterial communities in pupal samples.

10.1128/mSphere.00387-16.6 TABLE S3  Relative abundance (indicator value, ≥0.5; *P* ≥ 0.01) of bacterial taxa associated with developmental stages of *Culex nigripalpus* Theobald. Asterisks represent taxa that are either found in a lower proportion (i.e., <0.01 relative abundance per sample) or absent. Download TABLE S3, PDF file, 0.1 MB. Copyright © 2017 Duguma et al. 2017 Duguma et al. This content is distributed under the terms of the Creative Commons Attribution 4.0 International license.

### Bacterial communities in newly emerged *Culex nigripalpus* female adults.

Bacteria in newly emerged (<12 h after eclosion) non-blood-fed female adults reared from egg to adults outdoors were enriched with an unknown species (OTU 2) of *Comamondaceae* (20%), *Oleomonas* (7%), and *Arcobacter* (4.6%) (Table S3). *Wolbachia* was also found in 2 of the 14 adult samples, constituting 91% and <1% of their respective sequences but was absent in 12 other samples. These results suggest a likely inclusion of* Culex quinquefasciatus*, which is a known host of* Wolbachia* in those samples during DNA extraction. Other notable species were recovered at lower proportions (<1%) and include *Thorsellia anophelis*.

## DISCUSSION

### Effects of organic enrichments on microbial communities associated with *Culex nigripalpus*.

We tested the hypothesis that organic nutrient enrichment, a primary factor for eutrophication and pollution, would alter the microbial larval resources and thereby impact microbial communities associated with *Culex* disease vectors in replicated outdoor mesocosm experiments. Results of our study revealed that a significant increase in abundance of sestonic particles and planktonic microeukaryotes (i.e., ciliates, flagellates, and rotifers) in treatments with high-nutrient enrichments were in agreement with the bottom-up resource hypothesis: i.e., increasing nutrients will increase the abundance of both autotrophic and heterotrophic microorganisms (39, 40). This was further corroborated with the increase in chemical oxygen demand, a predictor of the amount of organic material available for oxidation, and microbial consumption in the high-nutrient treatments compared to low-nutrient treatments in this study. Total concentrations of nutrients increased immediately following the uncovering of the mesocosms, suggesting autotrophic and aerobic microbial colonization of the mesocosms.

Despite the apparent differences in microbiota and chemical variables in the water column (Fig. 1 to 3), microbial communities associated with mosquitoes developing in these two larval environments were not affected significantly. This could be due to a combination of factors, including food web-mediated factors, such as differences in the abundance of planktonic bacteriovores (i.e., flagellates, ciliates, and rotifers), and the remarkable variability in microbial communities among samples within each treatment group. Predation of bacteria by planktonic bacteriovores has been known to be intense (41), and these groups might have affected the bacterial diversity in the water column, a feeding zone of *Culex* mosquitoes. Alternatively, *Culex* larvae are considered omnivores, feeding on a variety of lower trophic microorganisms, including bacteria (16), and therefore the impact on bacterial diversity in the mosquitoes may not necessarily be affected by increases in bottom-up resources. A previous study of microbiota in other *Culex* mosquitoes sampled from different habitats with different nutrient concentrations (influenced by larval control treatments) also did not reveal significant differences in the bacterial communities in the larvae sampled from the different larval habitats (42–44).

Increases in nutrients such as nitrogen and phosphorus in aquatic habitats, as a result of runoff from agricultural practices and other nonpoint sources, have been reported to influence the abundance of disease vectors, particularly *Culex* mosquitoes that are more adapted to polluted environments (6, 8, 45, 46). In addition, organic enrichments have been shown to influence mosquito control strategies. For example, under high-organic-rich environments, the efficacy of a fungal biological control agent, *Lagenidium giganteum*, was significantly reduced (12). Other studies reported that increase in organic or inorganic mater in the water column negatively influenced the efficacy of the commonly used *Bacillus*-based larval control agents (13, 47). It is likely that the diverse microbial communities and sestonic particles ingested by mosquito larvae might provide immunity or protection of midgut epithelium that is considered a primary target of *Bacillus thuringiensis* serovar israelensis (Bti) toxins in polluted environments (13).

Mosquitoes, in general, and *Culex* mosquito vectors, in particular, are considered primary colonizers of newly created freshwater aquatic habitats and are well adapted to polluted environments (42, 46, 48). The difference in abundances of *C. nigripalpus* between treatments was not significant, suggesting that mosquito abundance was not influenced by water column nutrient concentrations, especially during the initial colonization of newly formed aquatic habitats. This study and others have shown that *C. nigripalpus* mosquitoes prefer to lay their eggs and develop in highly eutrophic habitats than their *C. quinquefasciatus* congeners (21, 23). *Culex quinquefasciatus* was considered to be the dominant species colonizer of eutrophic habitats (9, 24), but its abundance during the succession in our mesocosms was negligible compared to that of *C. nigripalpus*.

### Bacterial communities associated with different life stages of *Culex nigripalpus*.

Microbial communities associated with* C. nigripalpus* sampled during the autumn varied significantly among life stages from the same cohort. Bacterial communities from female adults, eggs, and pupae were dominated by *Alphabacteria* and *Betaproteobacteria*, whereas bacteria from larvae were dominated by *Arcobacter* (*Epsilonproteobacteria*), *Hydrogenophaga*, and *Agrobacterium* (*Alphaproteobacteria*), *Thorsellia* (*Gammaproteobacteria*), and* Clostridium* (*Firmicutes*). *Arcobacter* is ubiquitous in aquatic environments and, as a member of *Epsilonproteobacteria*, might be associated with sulfur cycling (49, 50). The mesocosms used in this study were filled with well water, which contains a relatively high sulfur concentration (~100 mg sulfate/liter [D. Duguma, unpublished data]). Nearly 92% *Arcobacter* sequences were from larvae, whereas very few were found associated with eggs (0.4%), pupae (2.8%), and adults (4.6%), suggesting that* Arcobacter* found associated with the mosquitoes in this study might be waterborne and thus ingested by the mosquito larvae. Several *Arcobacter* spp. are known to be pathogenic to humans and animals (51) and associated with polluted environments (52). Although we have not ruled out experimentally that this bacterium can be transstadially transmitted across life stages or is a pathogen, *Arcobacter* found associated with pupae and adult *C. nigripalpus* mosquitoes was likely ingested by the larvae from the water and passed down to pupae and adults. Considering that members of this genus of bacteria are recognized as emerging pathogens to humans and animals (51), the recovery of a relatively small proportion (e.g., 4.6%) of *Arcobacter* sequences in female adults suggests further study, including a possibility that these bacteria might be harbored in salivary glands, with a direct implication for pathogen transmission (53). Salivary glands have been shown to harbor diverse bacterial communities (53).

*Agrobacterium* OTUs were found in all samples, from egg to adult stages of *C. nigripalpus*, with the highest abundance found associated with the eggs. Members of this genus are known to transfer genetic materials between themselves and other eukaryotes, such as plants (54), and this genus was among the dominant genera recovered from *Aedes* mosquitoes (55). Although we rinsed the egg rafts multiple times with distilled water, it is possible that some of the communities found associated with eggs might have been unintentionally cosampled from the water surface during the sampling of the egg rafts. Future studies will investigate whether some of the communities found associated with eggs are obligate symbionts.

*Thorsellia anophelis* was also found in *C. nigripalpus*, with the greatest abundance of this bacterium found in pupae (11.0%) and in late instar larvae (10.5%). The abundance of this species was considerably lower in eggs (0.02%), early instars (0.5%), and adults (0.14%), corroborating previous studies that this symbiont is likely ingested by larvae and transferred to the subsequent developmental stages (43, 56–58). The dominance and persistence of *Thorsellia* spp. in life stages of *Culex* mosquitoes in this study and in previous studies (43, 44) and *Anopheles* (56) mosquitoes suggest a strong consideration for the development of paratransgenic mosquito control (59).

In conclusion, differences in environmental habitat variations might not affect the internal bacterial communities associated with *Culex* mosquito vectors, which instead may be influenced by seasonal variations. For the first time, we identified microbial communities associated with *C. nigripalpus* across developmental stages and identified potential candidates that will be further investigated for their role in bionomics and control of this mosquito species.

## MATERIALS AND METHODS

### Mesocosm experiment.

Our experimental design involved two contrasting larval environments in outdoor experimental mesocosms during autumn 2015. Two different larval environmental conditions were created on 27 October 2015 by adding two nutrient regimens: 0.2 and 1% (wt/vol) (low and high, respectively) rabbit food (alfalfa pellets) to three replicated outdoor mesocosms filled with 378 liters of well water at the University of Florida, Florida Medical Entomology Laboratory (see Fig. S4 in the supplemental material). The surface area and depth of water were 0.85 m^2^ and 0.5 m, respectively. Outdoor mesocosms can be used to examine various ecological hypotheses, including the effects of nutrients and climate change on aquatic food webs (18, 42, 43, 60, 61). Alfalfa-based organic matter is commonly used to attract egg-laying female mosquitoes and supports the production of *Culex* mosquitoes for longer periods of time (9, 38, 43, 62). The organic matter was allowed to ferment in the mesocosms for ~1 week while covered with a tarp. Natural oviposition by *Culex* mosquitoes occurred in all mesocosms <24 h after uncovering the mesocosms (i.e., on 2 November 2015). Two egg rafts likely laid by two female *Culex nigripalpus* mosquitoes from each of the six mesocosms were sampled on day 1 (3 November 2015). One egg raft laid by an individual mosquito sampled from each of the mesocosms was placed in modified BioQuip mosquito-rearing chambers (see Fig. S5 in the supplemental material) and then submerged in each of the six mesocosms to allow access to larval microbial food resources and development of these mosquitoes under the field conditions. The submerged portion of the device has screen meshes (300 nylon) built into each mosquito breeder (BioQuip, Inc., Rancho Dominguez, CA, USA) to allow access to larval resources in the water column, whereas the above water portion of the device captures adults emerging from the same cohort of eggs. The second egg raft taken from each of the mesocosms was taken to the laboratory, triple rinsed with distilled water, and aseptically cut into two halves. One-half of the rafts from each of the containers were preserved in 95% ethanol for DNA extraction, while the remaining halves were placed in 200 ml of distilled water in sterile plastic cups and then allowed to hatch in an environmental chamber at a temperature of 27°C for positive morphological identification of the larvae to *Culex nigripalpus*.

10.1128/mSphere.00387-16.7 FIG S4  Schematic sketch of outdoor mosquito-rearing mesocosms at the Florida Medical Entomology Laboratory. Mesocosms 1, 10, and 13 received high-nutrient enrichment, whereas 2, 9, and 14 received low-nutrient enrichment. Download FIG S4, PDF file, 0.2 MB. Copyright © 2017 Duguma et al. 2017 Duguma et al. This content is distributed under the terms of the Creative Commons Attribution 4.0 International license.

10.1128/mSphere.00387-16.8 FIG S5  Modified BioQuip environmental floating chamber for mosquito rearing under outdoor field conditions. Download FIG S5, PDF file, 0.2 MB. Copyright © 2017 Duguma et al. 2017 Duguma et al. This content is distributed under the terms of the Creative Commons Attribution 4.0 International license.

### Mosquito and water sampling.

Samples of two (early and late instars) *Culex nigripalpus* larval stages, pupae, and adult mosquitoes developed from the same egg rafts in the BioQuip rearing chambers were taken on different days (see Table S4 in the supplemental material), preserved in 95% ethanol, and stored at −20°C until DNA extraction. In addition, mosquito larval samples were taken in five 350-ml standard dips from each of the mesocosms at days 7 and 9 after the mesocosms were exposed to egg-laying female mosquitoes to determine the identity and abundance of mosquitoes found in the mesocosms.

10.1128/mSphere.00387-16.9 TABLE S4  Sampling schedule of *Culex nigripalpus* mosquitoes developing in outdoor aquatic mesocosms for DNA extraction. Column heads represent mesocosm identification numbers, with mesocosms 1, 3, and 10 receiving high-nutrient treatments and 2, 9, and 14 receiving low-nutrient-treatment regimens. Numbers 1 through 3 in cells indicate the number of mosquitoes or the half of the six egg rafts used for DNA extraction. Asterisks indicate two groups of 3 larvae that were sampled on those dates. —, sampling was not carried out due to lack of appropriate life stages of mosquitoes. Download TABLE S4, PDF file, 0.1 MB. Copyright © 2017 Duguma et al. 2017 Duguma et al. This content is distributed under the terms of the Creative Commons Attribution 4.0 International license.

Water samples were taken in 250-ml amber plastic bottles on days 2, 7, and 9 after mosquitoes colonized the mesocosms to determine total nitrogen, phosphorus, and chemical oxygen demand (COD) in the water column using a Hach DR3900 spectrophotometer (Hach Company, Loveland, CO). The water samples integrated both the surface water and ~10 cm below the surface of the water and were collected from the center of the mesocosm. Dissolved oxygen and pH in the water column were determined *in situ* using a YSI Professional Plus multiparameter instrument (YSI, Inc., Yellow Spring, OH). Temperature and light intensity in the mesocosms were monitored continuously using a Hobo Pendant temperature/light data logger (Onset Computer Corp., Bourne, MA).

Water samples were collected in duplicate 50-ml sterile centrifuge tubes on days 0, 4, and 9 after the mesocosms were opened and preserved with 1% Lugol’s iodine solution to quantify the abundance of microeukaryotes (i.e., ciliates, flagellates, and rotifers) found in the water column. The microeukaryotes were counted by direct microscopy on a hemocytometer using a Leica inverted microscope (Leica Microsystems, Inc., Buffalo Grove, IL). Particle size distributions for small (0.2- to 1.999-μm ESD) and large (2- to 60-μm ESD) particles that include both heterotrophic and autotrophic communities were determined using a Multisizer 4E Coulter Counter particle size analyzer (Beckman Coulter, Inc., Miami, FL) by a previously published procedure (42).

### DNA extraction, PCR, and MiSeq Illumina library preparation.

The general scheme of this study followed procedures described in previous studies (43, 63). Briefly, pooled DNA samples from 1 to 3 individuals from each of the life stages of *C. nigripalpus* were extracted using the DNeasy blood and tissue kit following the manufacturer’s protocol (Qiagen, Valencia, CA) in a laminar flow hood. Prior to DNA extraction, mosquitoes were surface sterilized with 95% ethanol and rinsed three times using molecular biology-grade UltraPure water (Quality Biological, Inc., Gaithersburg, MD). The samples were gently vortexed for 10 s in between rinsing. The mosquitoes were left to air dry under a laminar flow hood before extraction. Pooling of individual insects for microbial analyses have been used routinely in characterization of community profiles in insects (43, 44, 64). Pooling of individuals may have several advantages, including maximizing the sequence yield per sample (above negative controls) to discern microbial community differences between treatment samples in insects (64). We also extracted DNA from egg rafts to determine if there were maternally transmitted symbionts (e.g., *Wolbachia*) or unknown symbionts were present in this species. DNA from adult males was not extracted in this study because males have no known significance in transmitting pathogens.

The PCR procedures, sequence assembly, and analyses followed previous procedures described in other studies (65–67). Briefly, ~460-bp amplicons were generated using PCR from the V3 and V4 regions of 16S rRNA genes using Pro341F and Pro805R, which target both bacteria and archaea (68). Amplicons from each of the samples and replicate no-template controls were tagged with unique 6-base barcodes, amplified using Illumina-specific primers, and sequenced according to a previously established protocol (67) with some modification. The modification included a second round of PCR with 15 cycles for samples with low amplification on the first round of PCR. In brief all PCR products were combined and subjected to 250-bp end sequencing (Reagent kit v2, 500) on a MiSeq (Illumina, San Diego, CA).

### Data analysis.

Using AXIOME to manage sequences analysis (69), 16S rRNA gene reads were assembled by PANDAseq version 2.10 (70), with a quality threshold of 0.9 (which rejects sequences with low-quality scores), a minimum overlap of 10 bases, and a minimum assembled length of 100 bases, and sequences with ambiguous nucleotides were rejected. Operational taxonomic units (OTUs) were picked at 97% identity using the UPARSE algorithm USEARCH version 7.0.1090 (71) with *de novo* chimera checking. Taxonomic classification was performed on the representative sequence of each OTU using RDP version 2.2 (72) via QIIME (73), trained against the Greengenes (August 2013 revision) (74) reference set with a minimum posterior probability of 80%. Sequences were rarefied to the lowest number of sequences per sample (i.e., 43,611) for alpha and beta diversity analyses. To determine microbial community differences among mosquito samples originating from high- and low-nutrient treatments, principal coordinate analysis (PCoA) and nonmetric multidimensional scaling (NMDS) ordinations, based on the Bray-Curtis dissimilarity measures, were conducted using the vegan R package version 2.2-0 (75). In addition, a PCoA ordination based on UniFrac distance measures was carried out with QIIME to determine bacterial community differences among samples of multiple treatments and life stages. Multiresponse permutation procedures (MRPP) were used to test differences among sample groups based on distance measures. Core bacterial taxa were determined based on OTUs represented by at least one sequence per sample in all samples (76).

Repeated-measures analysis of variance (ANOVA) using JMP (77) was conducted to assess differences in environmental variables (e.g., nutrients, pH, dissolved oxygen, and microeukaryotes) and larval mosquito abundance in the water column between the two treatments. One-way ANOVA was performed to assess differences in mean abundance of total counts of small and large sestonic particles. Means were separated by Tukey’s test at *P* < 0.05, after performing Bonferroni correction on calculated *P* values.

### Accession number(s).

All sequence data for this study were submitted to the European Bioinformatics Institute under accession no. PRJEB17885.
