# Supplementary material for: Effects of Organic Amendments on Microbiota Associated with the Culex nigripalpus Mosquito Vector of the Saint Louis Encephalitis and West Nile Viruses
Source: mSphere. 2017 Feb 1;2(1):e00387-16. doi: 10.1128/mSphere.00387-16 (PMC5288567; doi:10.1128/mSphere.00387-16)
Supplement: TABLE S1 [file sph001172227st6.pdf]

Table S1

OTU_ID	cluster	Consensus Lineage	Phylum	Class	Order	Family	Genus	species	indicator value	*p*
X510	High	*Bacteria*	*Bacteroidetes*	*Bacteroidia*	*Bacteroidales*	*Porphyromonadacea*	*Dysgonomonas*		0.495	0.010
X9	High	*Bacteria*	*Firmicutes*	*Clostridia*	*Clostridiales*				0.832	0.020
X31	High	*Bacteria*	*Firmicutes*	*Clostridia*	*Clostridiales*	*Clostridiaceae*			0.784	0.010
X924	High	*Bacteria*	*Firmicutes*	*Clostridia*	*Clostridiales*	*Clostridiaceae*	*Clostridium*		0.697	0.000
X94	High	*Bacteria*	*Firmicutes*	*Clostridia*	*Clostridiales*	*Clostridiaceae*	*Clostridium*		0.673	0.000
X1277	High	*Bacteria*	*Firmicutes*	*Clostridia*	*Clostridiales*	*Clostridiaceae*	*Clostridium*		0.509	0.020
X1008	High	*Bacteria*	*Firmicutes*	*Clostridia*	*Clostridiales*	*Clostridiaceae*	*Clostridium*	*intestinale*	0.494	0.010
X1119	High	*Bacteria*	*Firmicutes*	*Clostridia*	*Clostridiales*	*Clostridiaceae*	*Clostridium*		0.494	0.020
X1109	High	*Bacteria*	*Firmicutes*	*Clostridia*	*Clostridiales*	*Clostridiaceae*			0.409	0.020
X170	Low	*Bacteria*	*Proteobacteria*	*Betaproteobacteria*	*Burkholderiales*	*Comamonadaceae*	*Variovorax*	*paradoxus*	0.480	0.010
X72	Low	*Bacteria*							0.447	0.030
X125	Low	*Bacteria*							0.427	0.010
X107	Low	*Bacteria*							0.405	0.010
