# Supplementary figures and images for: Effects of Organic Amendments on Microbiota Associated with the Culex nigripalpus Mosquito Vector of the Saint Louis Encephalitis and West Nile Viruses
Source: mSphere. 2017 Feb 1;2(1):e00387-16. doi: 10.1128/mSphere.00387-16 (PMC5288567; doi:10.1128/mSphere.00387-16)

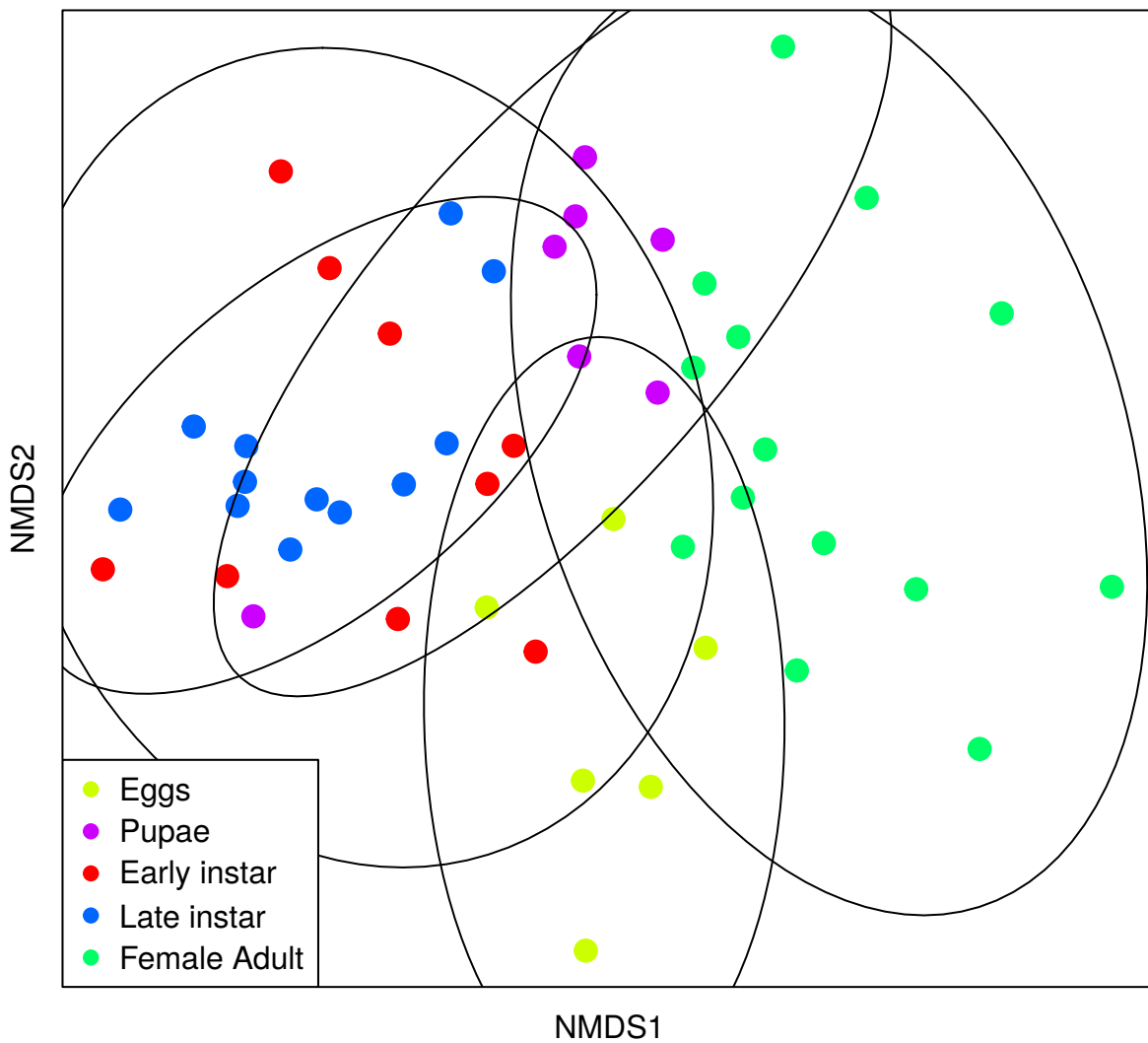

Supplement: FIG S2 [file sph001172227sf2.pdf]

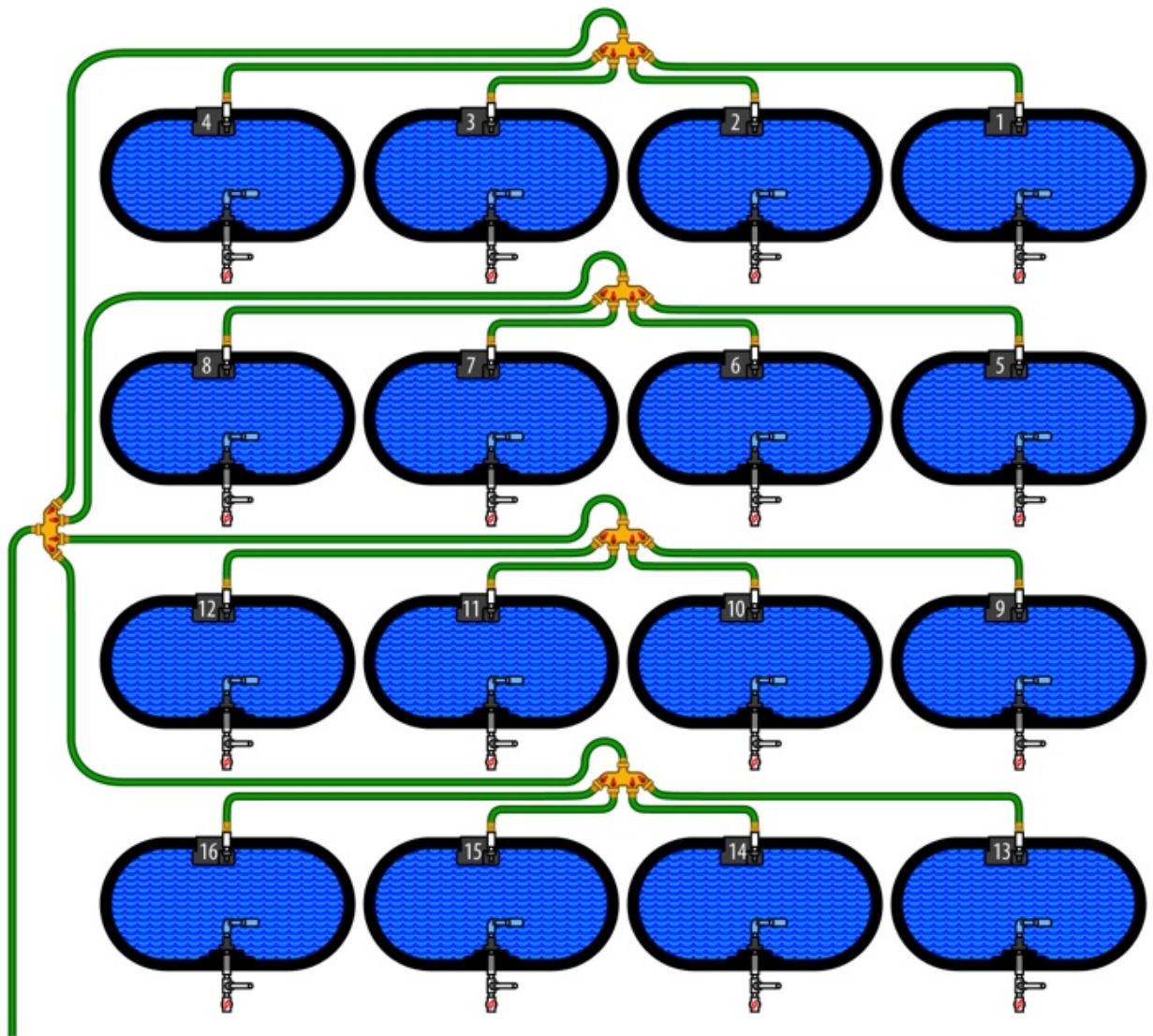

Supplement: FIG S4 [file sph001172227sf4.pdf]

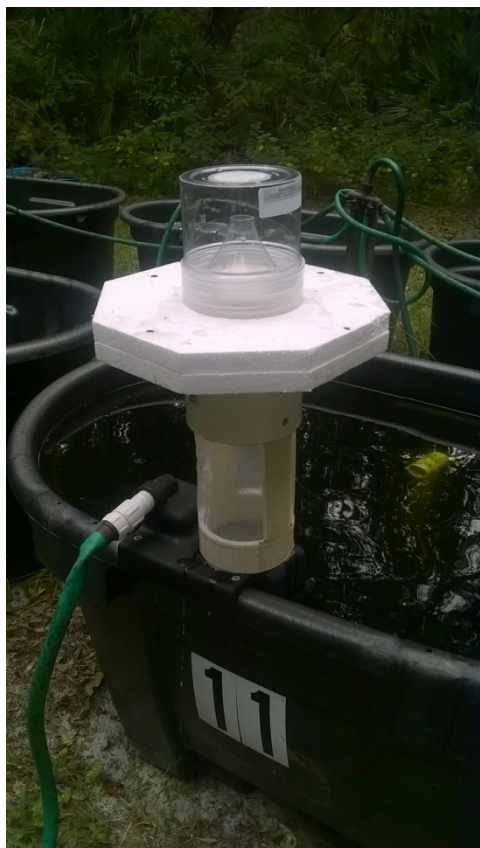

Supplement: FIG S5 [file sph001172227sf5.pdf]
