# Supplementary material for: Effects of Organic Amendments on Microbiota Associated with the Culex nigripalpus Mosquito Vector of the Saint Louis Encephalitis and West Nile Viruses
Source: mSphere. 2017 Feb 1;2(1):e00387-16. doi: 10.1128/mSphere.00387-16 (PMC5288567; doi:10.1128/mSphere.00387-16)
Supplement: TABLE S2 [file sph001172227st7.pdf]

Table S2

OUT_ID	cluster	Consensus Lineage	Phylum	Class	Order	Family	Genus	species	indicator_value	p
X7	eggs	Bacteria	Proteobacteria	Alphaproteobacteria	Rhodospirillales	Rhodospirillaceae	Oleomonas		0.850	0.001
X69	eggs	Bacteria	Proteobacteria	Betaproteobacteria	Burkholderiales	Comamonadaceae	Methylibium		0.750	0.019
X72	eggs	Bacteria							0.680	0.001
X1340	eggs	Unclassified							0.640	0.001
X1307	eggs	Bacteria	Proteobacteria	Betaproteobacteria	Burkholderiales	Comamonadaceae			0.610	0.006
X118	eggs	Unclassified							0.570	0.007
X107	eggs	Bacteria							0.540	0.003
X85	eggs	Bacteria	Proteobacteria	Betaproteobacteria	Burkholderiales	Comamonadaceae	Limnobacter		0.520	0.025
X1202	early instar	Bacteria	Actinobacteria	Actinobacteria	Actinomycetales				0.810	0.001
X34	early instar	Bacteria	Actinobacteria	Actinobacteria	Actinomycetales	Corynebacteriaceae	Corynebacterium		0.750	0.005
X79	early instar	Bacteria	Firmicutes	Bacilli	Lactobacillales	Enterococcaceae	Enterococcus	cecorum	0.740	0.005
X35	early instar	Bacteria	Firmicutes	Bacilli	Bacillales	Bacillaceae	Anoxybacillus	kestanbolensis	0.710	0.008
X33	early instar	Bacteria	Bacteroidetes	Flavobacteriia	Flavobacteriales	[Weeksellaceae]	Cloacibacterium		0.700	0.020
X148	early instar	Bacteria	Bacteroidetes	Flavobacteriia	Flavobacteriales	[Weeksellaceae]			0.670	0.003
X10	early instar	Bacteria	Proteobacteria	Alphaproteobacteria	Rhizobiales	Rhizobiaceae	Agrobacterium		0.660	0.046
X299	early instar	Bacteria	Proteobacteria	Betaproteobacteria	Burkholderiales	Alcaligenaceae	Achromobacter		0.580	0.001
X100	early instar	Bacteria	Firmicutes	Bacilli	Bacillales	[Thermicanaceae]	Thermicanus		0.550	0.014
X61	early instar	Bacteria	Proteobacteria	Betaproteobacteria	Burkholderiales	Comamonadaceae			0.530	0.024
X1111	early instar	Bacteria	Proteobacteria	Alphaproteobacteria	Rhizobiales				0.500	0.002
X1252	early instar	Bacteria							0.500	0.001
X1110	late instar	Bacteria	Proteobacteria	Alphaproteobacteria	Sphingomonadales	Sphingomonadaceae	Novosphingobium		0.990	0.001
X32	late instar	Bacteria	Bacteroidetes	Flavobacteriia	Flavobacteriales	Flavobacteriaceae	Flavobacterium		0.890	0.001
X1383	late instar	Bacteria	Proteobacteria	Alphaproteobacteria	Sphingomonadales				0.850	0.001
X49	late instar	Bacteria	Proteobacteria	Alphaproteobacteria	Sphingomonadales	Sphingomonadaceae	Novosphingobium		0.840	0.003
X88	late instar	Bacteria	Proteobacteria	Alphaproteobacteria	Sphingomonadales	Sphingomonadaceae	Novosphingobium		0.830	0.001
X18	late instar	Bacteria	Proteobacteria	Betaproteobacteria	Burkholderiales	Comamonadaceae	Hydrogenophaga		0.820	0.001
X652	late instar	Bacteria	Proteobacteria	Alphaproteobacteria	Sphingomonadales	Sphingomonadaceae	Novosphingobium		0.790	0.001
X26	late instar	Bacteria	Proteobacteria	Betaproteobacteria	Rhodocyclales	Rhodocyclaceae			0.780	0.006
X97	late instar	Bacteria	Proteobacteria	Alphaproteobacteria	Caulobacterales	Caulobacteraceae			0.770	0.001
X186	late instar	Bacteria	Proteobacteria	Betaproteobacteria	Rhodocyclales	Rhodocyclaceae	Uliginosibacterium		0.710	0.002
X39	late instar	Bacteria	Bacteroidetes	[Saprospirae]	[Saprospirales]	Chitinophagaceae			0.700	0.002
X858	late instar	Bacteria	Proteobacteria	Betaproteobacteria	Burkholderiales	Comamonadaceae			0.680	0.002
X1442	late instar	Bacteria	Proteobacteria	Betaproteobacteria	Rhodocyclales	Rhodocyclaceae	Dechloromonas	fungiphilus	0.680	0.001
X479	late instar	Bacteria	Bacteroidetes	Flavobacteriia	Flavobacteriales	Cryomorphaceae	Fluviicola		0.680	0.002
X1205	late instar	Bacteria	Proteobacteria	Alphaproteobacteria	Rhodobacterales	Rhodobacteraceae	Rhodobacter		0.660	0.039
X52	late instar	Bacteria	Proteobacteria	Alphaproteobacteria	Rhizobiales				0.650	0.016
X1537	late instar	Bacteria	Proteobacteria	Betaproteobacteria	Burkholderiales	Comamonadaceae	Hydrogenophaga		0.640	0.003
X93	late instar	Bacteria	Proteobacteria	Alphaproteobacteria	Rhodobacterales	Rhodobacteraceae	Rhodobacter		0.640	0.006
X861	late instar	Bacteria	Proteobacteria	Gammaproteobacteria	Alteromonadales	[Chromatiaceae]	Rheinheimera		0.600	0.005
X98	late instar	Bacteria	Proteobacteria	Alphaproteobacteria	Sphingomonadales	Erythrobacteraceae			0.570	0.005
X41	late instar	Bacteria	Proteobacteria	Alphaproteobacteria	Sphingomonadales	Sphingomonadaceae	Blastomonas		0.560	0.010
X138	late instar	Bacteria	SR1						0.560	0.006
X313	late instar	Bacteria	Bacteroidetes	Cytophagia	Cytophagales	Cytophagaceae	Runella		0.550	0.001
X1193	late instar	Bacteria	Proteobacteria	Betaproteobacteria	Burkholderiales	Comamonadaceae			0.500	0.011
X29	pupae	Bacteria	Bacteroidetes	Cytophagia	Cytophagales	Cytophagaceae	Flectobacillus		0.570	0.029
X1008	pupae	Bacteria	Firmicutes	Clostridia	Clostridiales	Clostridiaceae	Clostridium	intestinale	0.540	0.008
X258	pupae	Bacteria	Bacteroidetes	Bacteroidia	Bacteroidales	Porphyromonadaceae	Paludibacter		0.510	0.004
X68	female adult	Bacteria	Proteobacteria	Alphaproteobacteria	Rhizobiales				0.830	0.001
X45	female adult	Bacteria	Proteobacteria	Betaproteobacteria	Burkholderiales	Comamonadaceae	Hydrogenophaga		0.760	0.001
X236	female adult	Bacteria	Firmicutes	Clostridia	Clostridiales				0.750	0.001
X20	female adult	Bacteria	Proteobacteria	Betaproteobacteria	Rhodocyclales	Rhodocyclaceae	Dechloromonas		0.650	0.024
X127	female adult	Bacteria	Proteobacteria	Alphaproteobacteria	Rhizobiales	Hyphomicrobiaceae	Rhodoplanes	elegans	0.600	0.003
X448	female adult	Bacteria	Firmicutes	Clostridia	Clostridiales	Clostridiaceae	Clostridium		0.600	0.008
X3	female adult	Bacteria	Tenericutes	Mollicutes	RsaHF231				0.580	0.025
X586	female adult	Bacteria	Firmicutes	Clostridia	Clostridiales	Veillonellaceae			0.570	0.006
X37	female adult	Bacteria	Firmicutes	Clostridia	Clostridiales	Clostridiaceae	Clostridium		0.570	0.012
X167	female adult	Bacteria	Firmicutes	Clostridia	Clostridiales	Peptococcaceae	Desulfosporosinus	meridiei	0.550	0.007
X95	female adult	Bacteria	Firmicutes	Clostridia	Clostridiales	Clostridiaceae	Clostridium		0.530	0.038
X523	female adult	Bacteria	Firmicutes	Clostridia	Clostridiales	Peptococcaceae	Desulfosporosinus	meridiei	0.530	0.004
X139	female adult	Bacteria	Firmicutes	Clostridia	Clostridiales	Lachnospiraceae	Coprococcus		0.510	0.011
X696	female adult	Bacteria	Firmicutes	Clostridia	Clostridiales	Veillonellaceae			0.500	0.001
X1173	female adult	Bacteria	Firmicutes	Bacilli	Lactobacillales	Enterococcaceae	Enterococcus		0.500	0.001
X820	female adult	Bacteria	Firmicutes	Clostridia	Clostridiales	Veillonellaceae			0.500	0.003
