# Supplementary material for: Effects of Organic Amendments on Microbiota Associated with the Culex nigripalpus Mosquito Vector of the Saint Louis Encephalitis and West Nile Viruses
Source: mSphere. 2017 Feb 1;2(1):e00387-16. doi: 10.1128/mSphere.00387-16 (PMC5288567; doi:10.1128/mSphere.00387-16)
Supplement: TABLE S3 [file sph001172227st8.pdf]

Table S3

Phylum	Class	Order	Family	Genus	Relative abundance (proportion)				
					eggs	early instars	late instar	pupae	adults
Acidobacteria	*Holophagae*	*Holophagales*	*Holophagaceae*		*	*	*	*	*
Actinobacteria	*Actinobacteria*	*Actinomycetales*	*Corynebacteriaceae*	*Corynebacterium*	0.056	*	*	*	0.018
Actinobacteria	*Actinobacteria*	*Actinomycetales*			0.014	*	*	*	*
Actinobacteria	*Actinobacteria*	*Actinomycetales*	*Propionibacteriaceae*	*Propionibacterium*	0.012	*	*	*	0.010
Bacteria					*	*	*	*	0.084
Bacteroidetes	*[Saprospirae]*	*[Saprospirales]*	*Chitinophagaceae*		*	*	*	0.023	*
Bacteroidetes	*Bacteroidia*	*Bacteroidales*			*	0.010	0.014	*	*
Bacteroidetes	*Cytophagia*	*Cytophagales*	*Cytophagaceae*	*Flectobacillus*	*	0.020	*	*	*
Bacteroidetes	*Flavobacteriia*	*Flavobacteriales*	*[Weeksellaceae]*	*Cloacibacterium*	0.055	*	*	*	*
Bacteroidetes	*Flavobacteriia*	*Flavobacteriales*	*Flavobacteriaceae*	*Flavobacterium*	*	*	*	0.027	*
Firmicutes	*Bacilli*	*Bacillales*	*Bacillaceae*	*Anoxybacillus*	0.022	*	*	*	*
Firmicutes	*Bacilli*	*Bacillales*	*Staphylococcaceae*	*Staphylococcus*	*	*	*	*	0.021
Firmicutes	*Bacilli*	*Lactobacillales*	*Enterococcaceae*	*Enterococcus*	0.039	*	*	*	*
Firmicutes	*Clostridia*	*Clostridiales*	*Clostridiaceae*	*Clostridium*	0.015	0.040	0.051	0.014	*
Firmicutes	*Clostridia*	*Clostridiales*	*Clostridiaceae*		0.023	0.030	0.038	0.019	*
Firmicutes	*Clostridia*	*Clostridiales*			*	0.020	0.059	*	0.013
Firmicutes	*Clostridia*	*Clostridiales*	*Ruminococcaceae*		*	0.050	0.024	*	*
Firmicutes	*Erysipelotrichi*	*Erysipelotrichales*	*Erysipelotrichaceae*		*	0.010	*	*	*
Firmicutes	*Erysipelotrichi*	*Erysipelotrichales*	*Erysipelotrichaceae*		*	0.020	0.028	0.026	*
Unclassfied					*	*	*	*	0.011
Proteobacteria	*Alphaproteobacteria*	*Rhizobiales*			*	*	0.016	0.034	*
Proteobacteria	*Alphaproteobacteria*	*Rhizobiales*	*Rhizobiaceae*	*Agrobacterium*	0.125	0.010	0.010	*	*
Proteobacteria	*Alphaproteobacteria*	*Rhizobiales*	*Rhizobiaceae*		0.015	0.020	*	*	*
Proteobacteria	*Alphaproteobacteria*	*Rhodobacterales*	*Rhodobacteraceae*	*Rhodobacter*	*	0.010	*	0.031	0.017
Proteobacteria	*Alphaproteobacteria*	*Rhodospirillales*	*Rhodospirillaceae*	*Oleomonas*	*	*	*	*	0.069
Proteobacteria	*Alphaproteobacteria*	*Rickettsiales*	*Rickettsiaceae*	*Wolbachia*	*	*	*	*	0.066
Proteobacteria	*Alphaproteobacteria*	*Sphingomonadales*	*Sphingomonadaceae*	*Blastomonas*	*	*	*	0.011	*
Proteobacteria	*Alphaproteobacteria*	*Sphingomonadales*	*Sphingomonadaceae*	*Novosphingobium*	*	*	*	0.059	*
Proteobacteria	*Betaproteobacteria*	*Burkholderiales*	*Comamonadaceae*	*Comamonas*	0.022	0.010	*	*	0.018
Proteobacteria	*Betaproteobacteria*	*Burkholderiales*	*Comamonadaceae*	*Hydrogenophaga*	*	*	0.021	0.137	0.020
Proteobacteria	*Betaproteobacteria*	*Burkholderiales*	*Comamonadaceae*	*Limnohabitans*	*	*	*	0.061	*
Proteobacteria	*Betaproteobacteria*	*Burkholderiales*	*Comamonadaceae*		0.354	0.140	0.029	0.099	0.196
Proteobacteria	*Betaproteobacteria*	*Methylophilales*	*Methylophilaceae*	*Methylotenera*	*	*	*	*	0.020
Proteobacteria	*Betaproteobacteria*	*Neisseriales*	*Neisseriaceae*	*Vogesella*	*	0.060	0.026	*	*
Proteobacteria	*Betaproteobacteria*	*Rhodocyclales*	*Rhodocyclaceae*	*Dechloromonas*	*	*	0.054	0.036	*
Proteobacteria	*Betaproteobacteria*	*Rhodocyclales*	*Rhodocyclaceae*		*	*	*	0.051	*
Proteobacteria	*Epsilonproteobacteria*	*Campylobacterales*	*Campylobacteraceae*	*Arcobacter*	0.005	0.310	0.265	0.034	0.037
Proteobacteria	*Gammaproteobacteria*	*Aeromonadales*	*Aeromonadaceae*		0.013	*	*	*	0.036
Proteobacteria	*Gammaproteobacteria*	*Alteromonadales*	*[Chromatiaceae]*	*Rheinheimera*	*	*	*	0.017	*
Proteobacteria	*Gammaproteobacteria*	*Enterobacteriales*	*Enterobacteriaceae*		*	0.030	*	*	*
Proteobacteria	*Gammaproteobacteria*	*Enterobacteriales*	*Enterobacteriaceae*	*Providencia*	*	*	*	*	0.012
Proteobacteria	*Gammaproteobacteria*	*Enterobacteriales*	*Enterobacteriaceae*	*Thorsellia*	*	*	0.105	0.110	*
Proteobacteria	*Gammaproteobacteria*	*Pseudomonadales*	*Moraxellaceae*	*Acinetobacter*	*	*	*	*	0.021
Proteobacteria	*Gammaproteobacteria*	*Pseudomonadales*	*Pseudomonadaceae*		0.012	*	*	*	0.029
Tenericutes	*Mollicutes*	*RsaHF231*			*	0.040	0.103	0.027	*
