# Supplementary material for: Effects of Organic Amendments on Microbiota Associated with the Culex nigripalpus Mosquito Vector of the Saint Louis Encephalitis and West Nile Viruses
Source: mSphere. 2017 Feb 1;2(1):e00387-16. doi: 10.1128/mSphere.00387-16 (PMC5288567; doi:10.1128/mSphere.00387-16)
Supplement: TABLE S4 [file sph001172227st9.pdf]

Table S4. Sampling schedule of *Culex nigripalpus* mosquitoes developing in outdoor aquatic mesocosms for DNA extraction. Column heads represent mesocosm identification numbers with 1, 10 and 13 received high nutrient treatments and mesocosms 2, 9 and 14 received low treatment regimens. Numbers (1–3) in cells indicate the number of mosquitoes or the half of the six egg rafts used for DNA extraction. The "*" indicates two groups of 3 larvae that were sampled on those dates. The "—" designated that sampling was not carried out due to lack of appropriate life stages of mosquitoes.

Stage	Time (Day)	Sampling date	1	2	9	10	13	14
eggs	0	11/3/15	½	½	½	½	½	½
early instar	1	11/4/15	2	3	3	3	3	3
early instar	4	11/7/15	—	—	3	—	3	—
late instar	4	11/7/15	3	3	–	3	—	3*
late instar	6	11/9/15	—	—	3	3	3	—
pupae	6	11/9/15	3*	3*	3*	—	—	—
female adult	6	11/9/15	—	—	—	—	—	3
female adult	8	11/11/15	—	3	3	—	—	2
early instar	9	11/12/15	—	—	—	—	—	3
female adult	9	11/12/15	—	2	3	—	—	1
late instar	9	11/12/15	3	3	—	3	3	—
pupae	9	11/12/15	—	—	—	—	—	3
female adult	10	11/13/15	—	3	3	—	—	—
female adult	11	11/14/15	—	—	—	1	–	—
female adult	17	11/20/15	1	—	—	—	1	—
female adult	20	11/23/15	—	—	—	3	3	—
